# Acknowledgments

**DOI:** 10.1002/jgf2.287

**Published:** 2019-11-24

It would not be possible to edit the Journal without the advice of many experts. The Editor is grateful to the following people, and members of the Editorial Board, each of whom has reviewed from one to many manuscripts for *Journal of General and Family Medicine* during the past year.

Abe, Toshikazu

Asakura, Kentaro

Azuma, Teruhisa

Dohi, Eisuke

Endo, Takeshi

Funakoshi, Hiraku

Gibo, Koichiro

Gomi, Harumi

Gotoh, Jun

Hagiya, Hideharu

Hamada, Toshihisa

Hamaguchi, Sugihiro

Harada, Taku

Haruta, Junji

Hasegawa, Hideki

Hasegawa, Yukihito

Hashimoto, Kunihiko

Hashizume, Naoki

Hayashi, Tetsuro

Hayashi, Tsuneari

Hirayama, Yoko

Hojo, Mariko

Honda, Hitoshi

Horibata, Ken

Horikoshi, Yuho

Ichikawa, Hiroko

Ie, Kenya

Iga, Toru

Ikemoto, Tatsunori

Inoue, Kazuo

Ishikawa, Yukiko

Ishimaru, Hiroyasu

Iwamuro, Masaya

Kajihara, Yusaku

Kakeya , Hiroshi

Kameoka, Junichi

Kaneko, Makoto

Kaneko, Masahiro

Kanke, Satoshi

Kashima, Saori

Kashiwagi, Shinichiro

Kataoka, Yuki

Katsuki, Naoko

Kawamoto, Ryuichi

Kawasaki, Tatsuya

Kawashima, Atsushi

Kenzaka, Tsuneaki

Kimura, Takuma

Kinjo, Mitsuyo

Kita, Keiichiro

Kobayashi, Hiroyuki

Komatsu, Takayuki

Kosaka, Shintaro

Koyama, Hiroshi

Kume, Yu

Kurasawa, Gotaro

Kutsuna, Satoshi

Maeda, keisuke

Masoudi, Reza

Matsukawa, Noriyuki

Matsumura, Shinji

Matsuo, Hiroo

Matsuo, Takahiro

Matsushita, Masahide

Matsuyama, Yasushi

Mitsuhashi, Toshiharu

Miyazaki, Kei

Mizuno, Atsushi

Mori, Takahiro

Motomura, Kazuhisa

Nagai, Yuki

Nagata, Eiichiro

Nakamura, Tsukasa

Nakano, Toshiaki

Nakata , Hirotomo

Nakayama, Kuniko

Nango, Eishu

Narita, Masashi

Narumoto, Keiichiro

Nishioka, Hiroaki

Nishioka, Hiroaki

Nureki, Shin‐ichi

Ohira, Yoshiyuki

Ohishi, Tsuyoshi

Ohji , Goh

Ohta, Ryuichi

Oku, Tomohisa

Otaka, Shunichi

Ozaki, Nobuaki

Ozone, Sachiko

Rokutanda, Ryo

Saiki, Takuya

Sairenji , Tomoko

Sakamoto, So

Sakamoto, Yuichi

Sakata, Yasuhisa

Saraya, Takeshi

Sasaki, Yosuke

Satoi, Yoshinao

Shimajima, Yasuhiro

Shimizu, Ikuo

Shimizu, Keiki

Shimizu, Taro

Shimoda, Shinji

Sugiyama, Kemmyo

Takahashi, Fumihiko

Takahashi, Noriyuki

Takamura, Akiteru

Takeshima, Taro

Tanaka, Junichi

Taniguchi , Satoshi

Taniguchi, Toshibumi

Tanoue, Hiroki

Tokuda, Koichi

Tsuyuguchi, Toshio

Ui, Mutsuhito

Ukawa, Shigekazu

Wada, Mikio

Watanabe, Takamasa

Watari, Takashi

Wijeratne, T.

Yahata, Shinsuke

Yamamori, Ikuo

Yamamoto, Shungo

Yamamoto, Shunji

Yamamoto, Yu

Yanagi, Hidetaka

Yodoshi, Toshifumi

Yokokawa, Daiki

Yokokawa, Hirohide

Yoshida, Eriko

Yoshida, Shuhei

